# Supplementary material for: Design of ultra-swollen lipidic mesophases for the crystallization of membrane proteins with large extracellular domains
Source: Nat Commun. 2018 Feb 7;9:544. doi: 10.1038/s41467-018-02996-5 (PMC5803273; doi:10.1038/s41467-018-02996-5)
Supplement: Supplementary file 1 — Supplementary Information [file 41467_2018_2996_MOESM1_ESM.pdf]

Supplementary Information

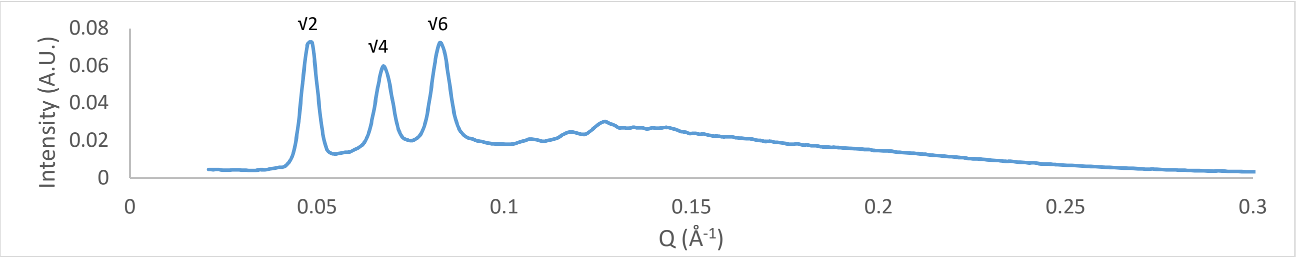

(a) 3%DSPG/MP 60%H2O

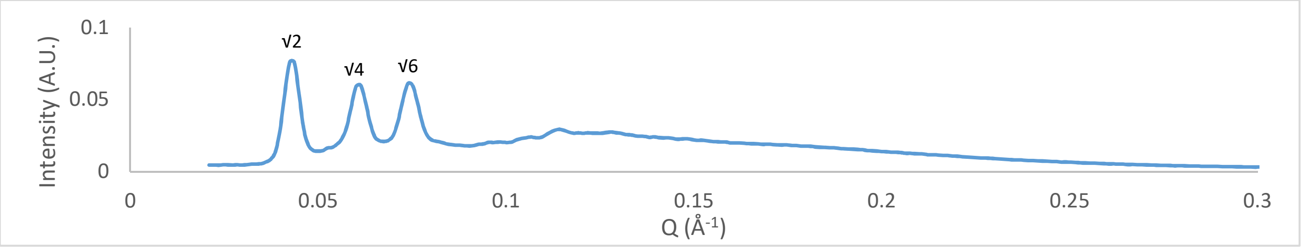

(b) 3%DSPG/MP 65%H2O

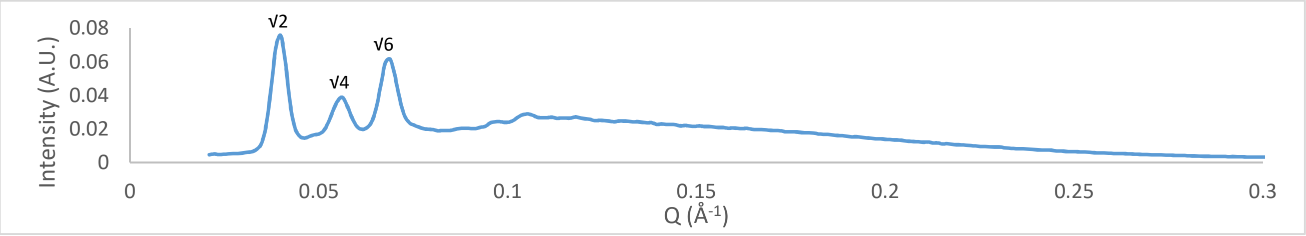

(c) 3%DSPG/MP 70%H2O

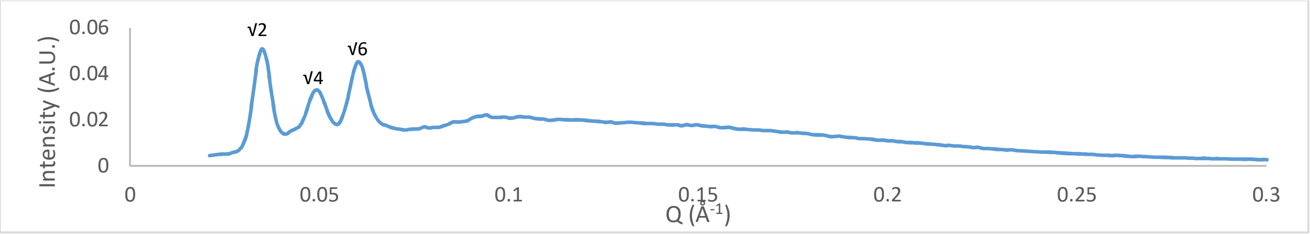

(d) 3%DSPG/MP 75%H2O

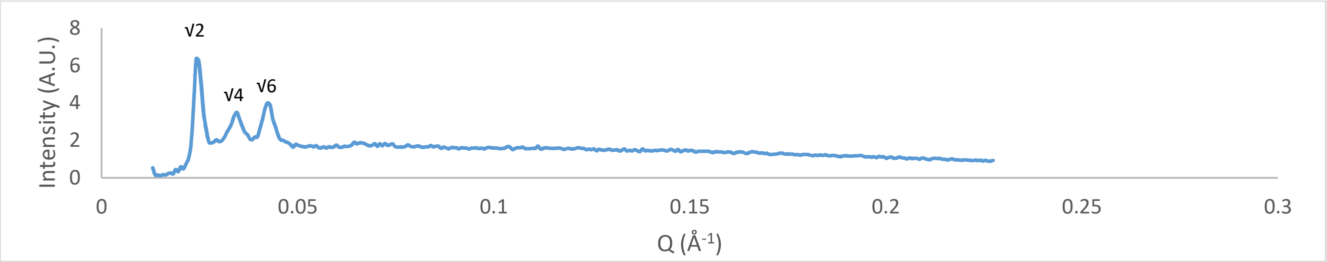

(e) 3%DSPG/MP 80%H2O

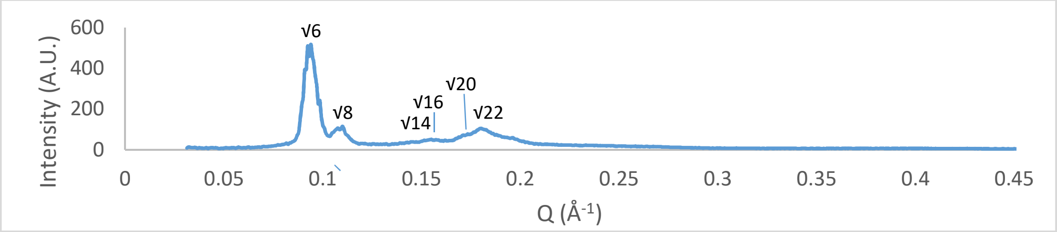

(f) 5%DSPG/MP 40%H2O

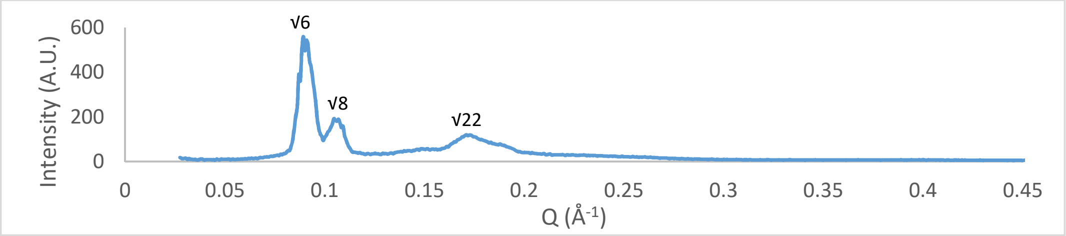

(g) 5%DSPG/MP 45%H2O

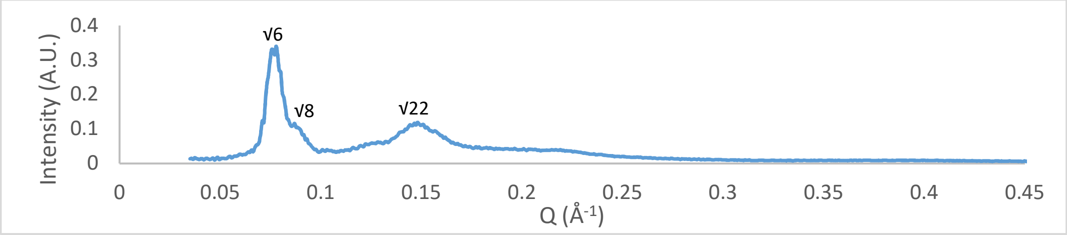

(h) 5%DSPG/MP 50%H2O

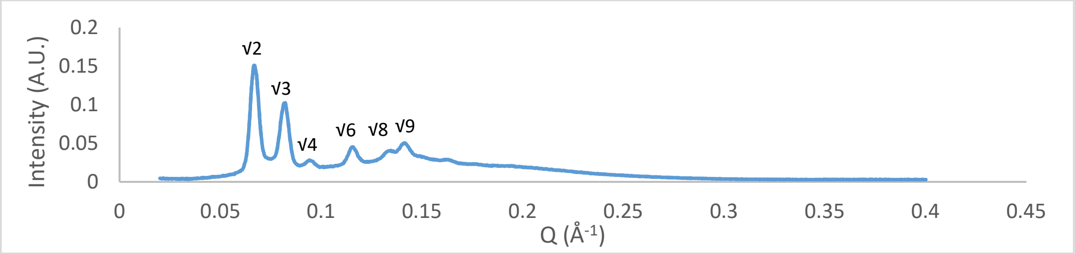

(i) 5%DSPG/MP 55%H2O

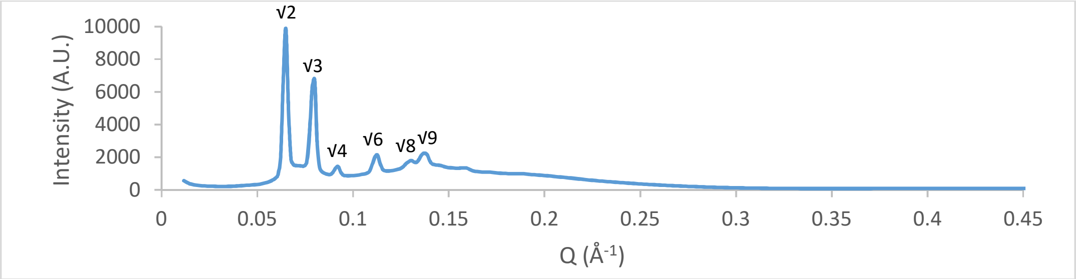

(j) 5%DSPG/MP 60%H2O

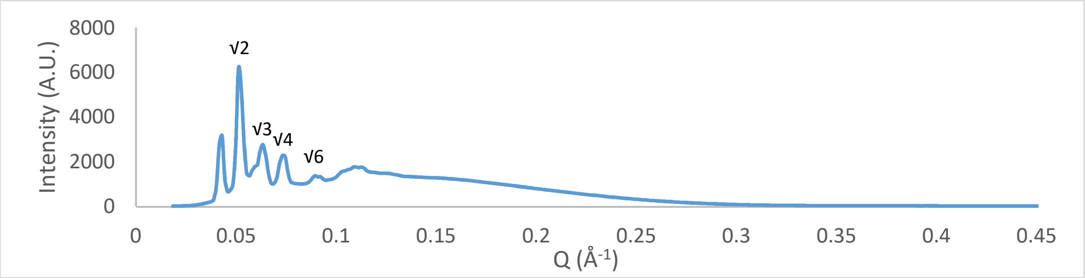

(k) 5%DSPG/MP 65%H2O (mix phase)

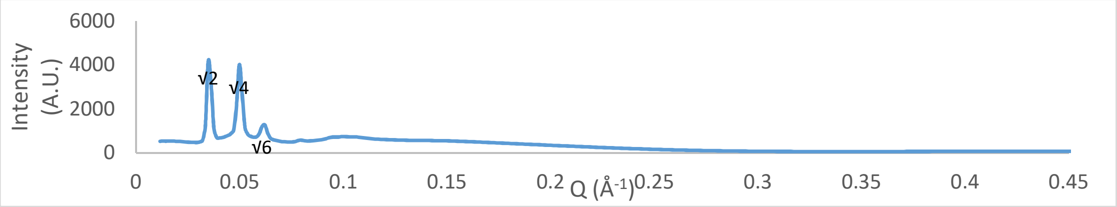

(l) 5%DSPG/MP 70%H2O (mix phase)

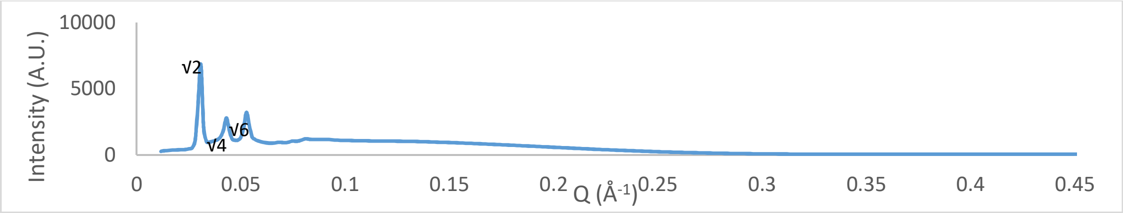

(m) 5%DSPG/MP 75%H2O

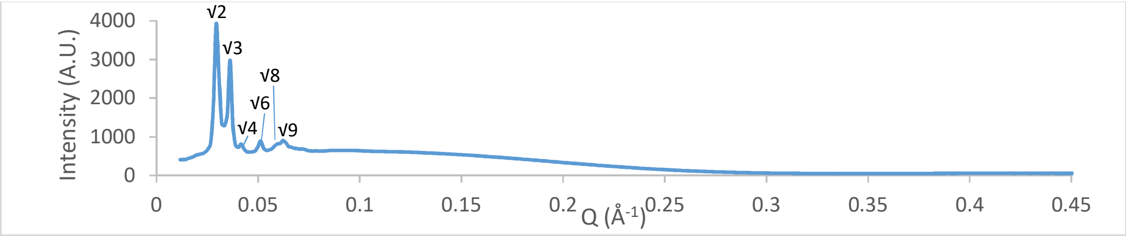

(n) 5%DSPG/MP 80%H2O

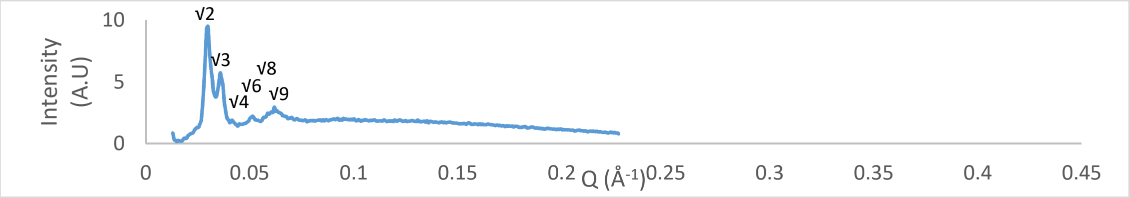

(o) 6%DSPG/MP 80%H2O

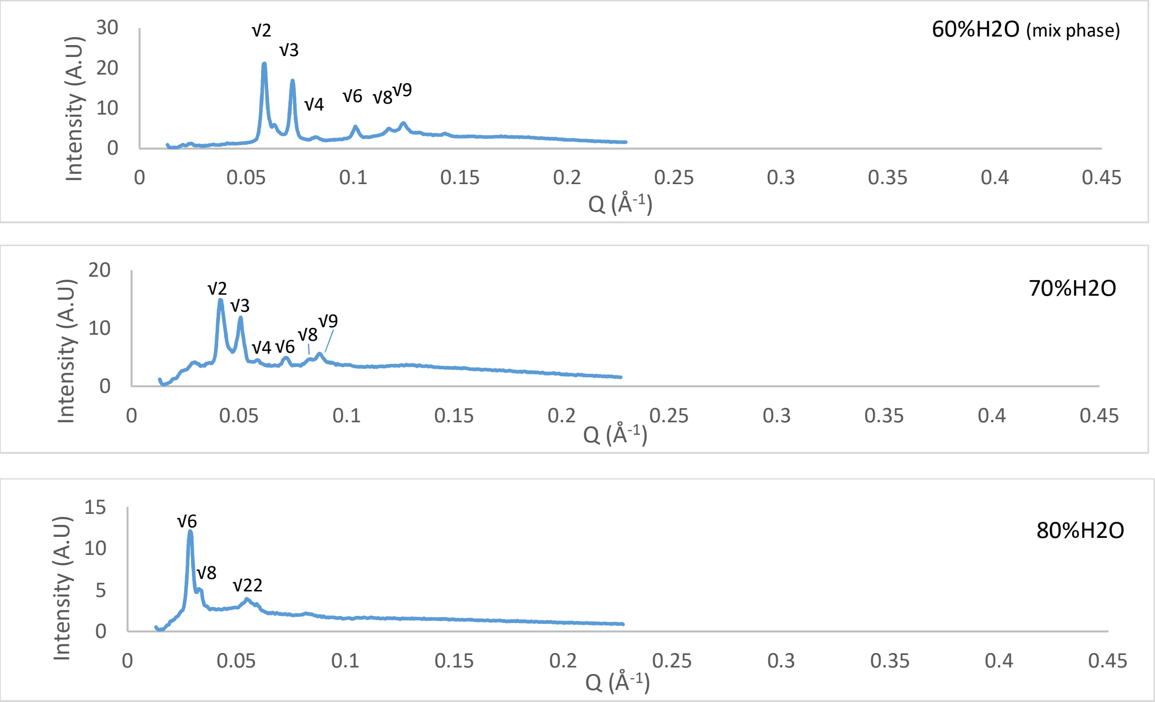

(p) 7%DSPG/MP

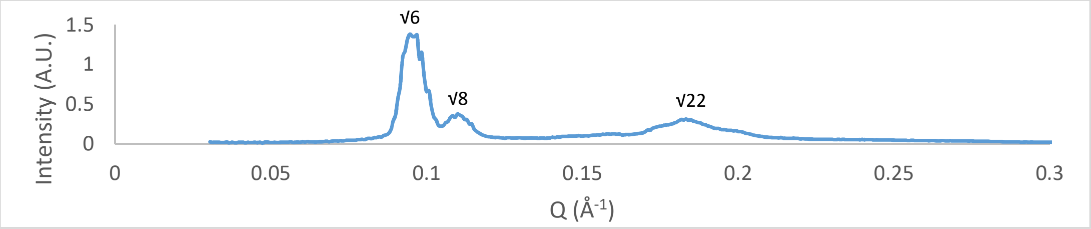

(q) 8%DSPG/MP 40%H2O

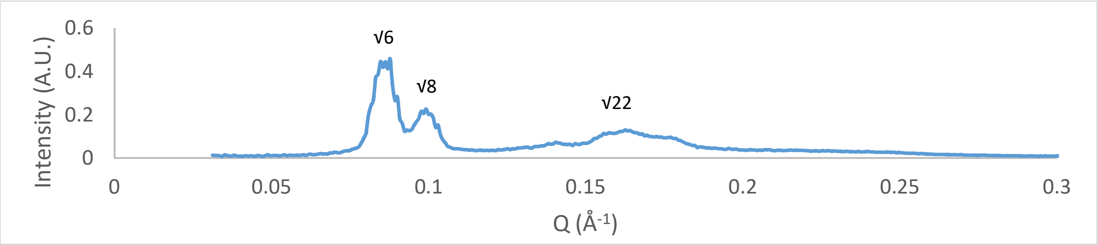

(r) 8%DSPG/MP 45%H2O

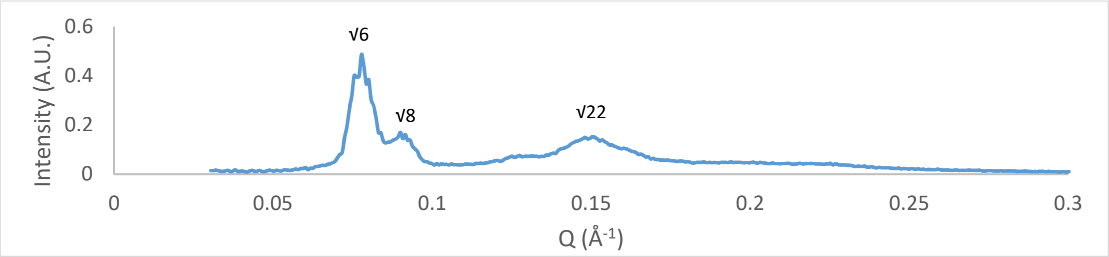

(s) 8%DSPG/MP 50%H2O

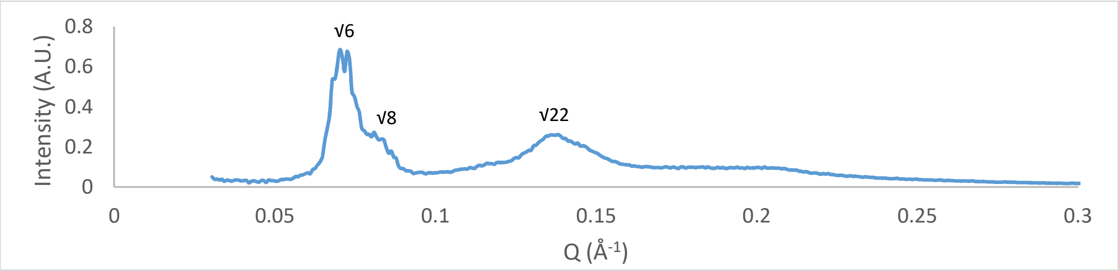

(t) 8%DSPG/MP 55%H2O

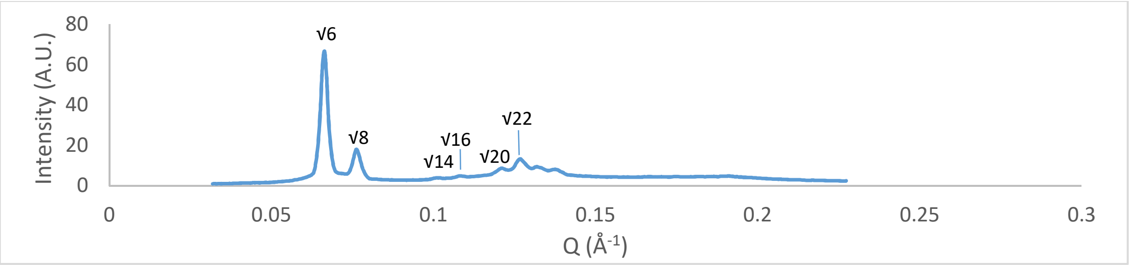

(u) 8%DSPG/MP 60%H2O

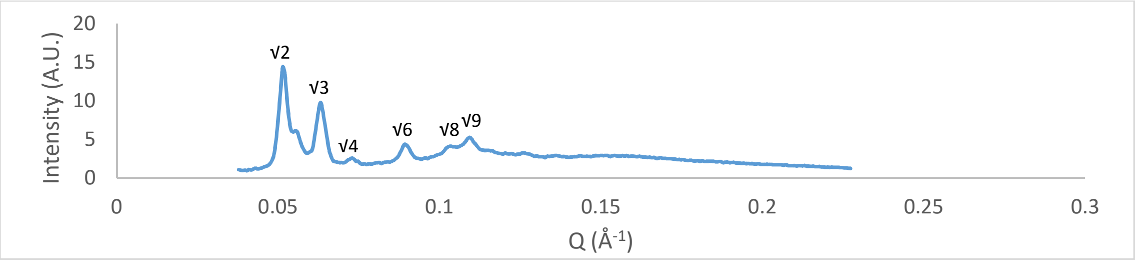

(v) 8%DSPG/MP 65%H2O (mix phase)

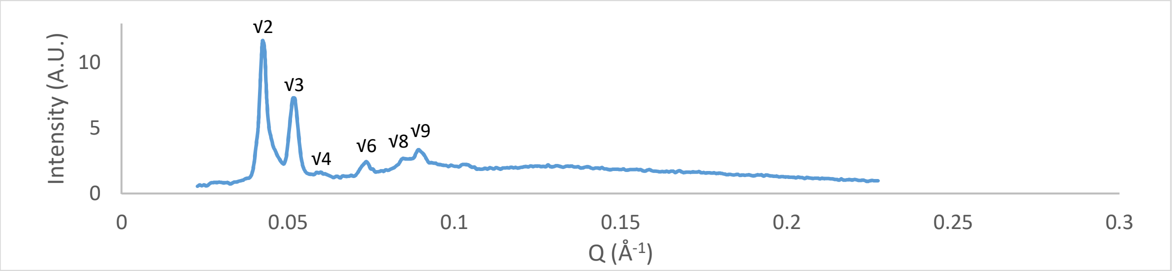

(w) 8%DSPG/MP 70%H2O

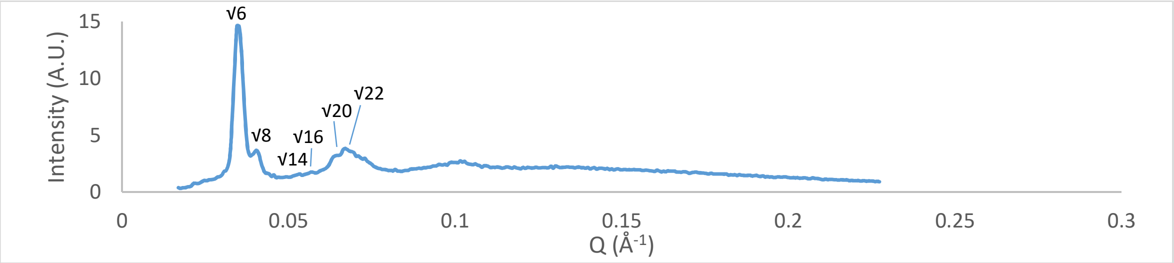

(x) 8%DSPG/MP 75%H2O

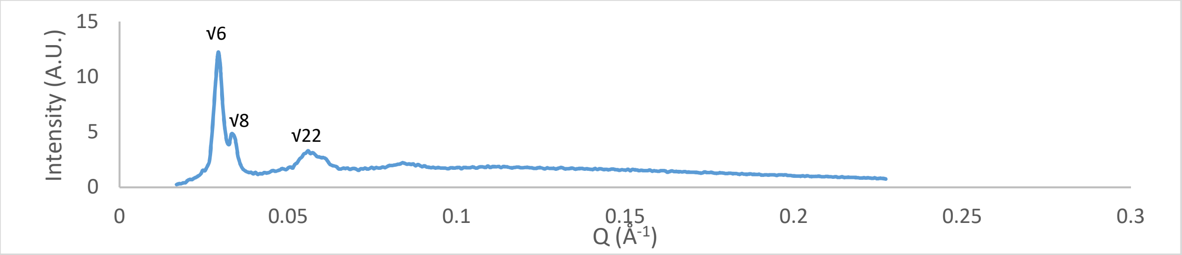

(y) 8%DSPG/MP 80%H2O

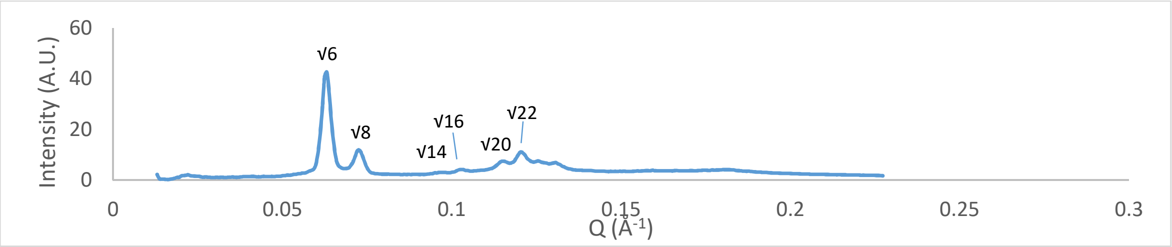

(z) 9%DSPG/MP 60%H2O

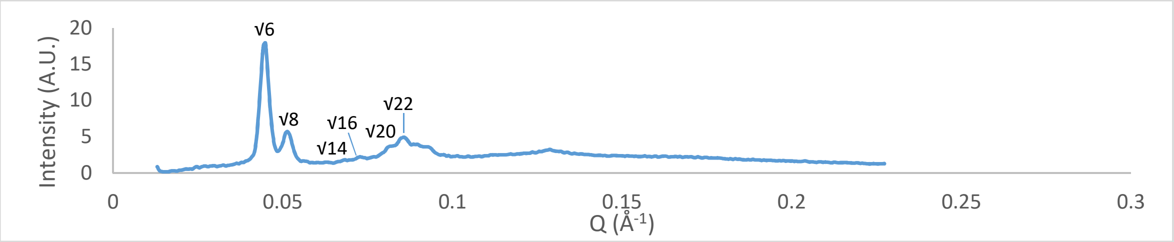

(aa) 9%DSPG/MP 70%H2O

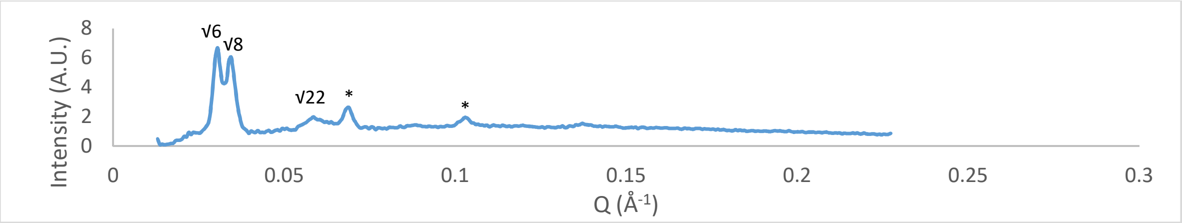

(ab) 9%DSPG/MP 80%H2O (mix phase: L_α(*))

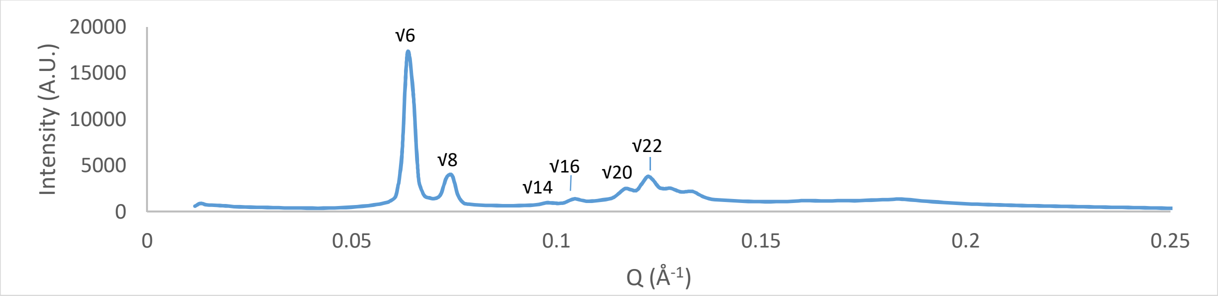

(ac) 10%DSPG/MP 60%H2O

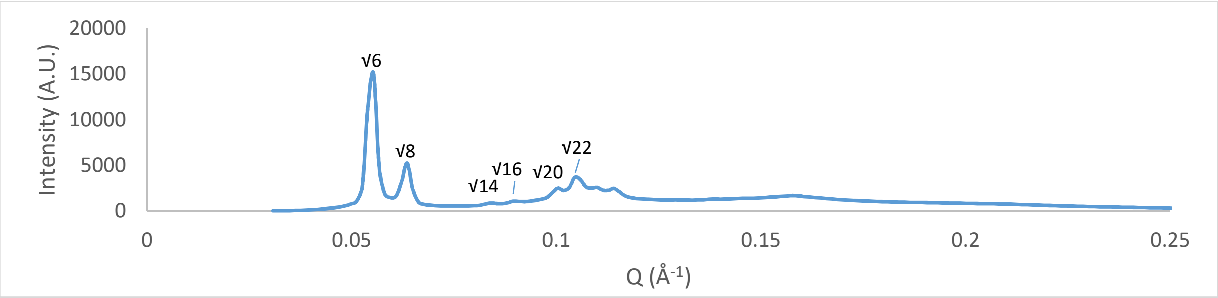

(ad) 10%DSPG/MP 65%H2O

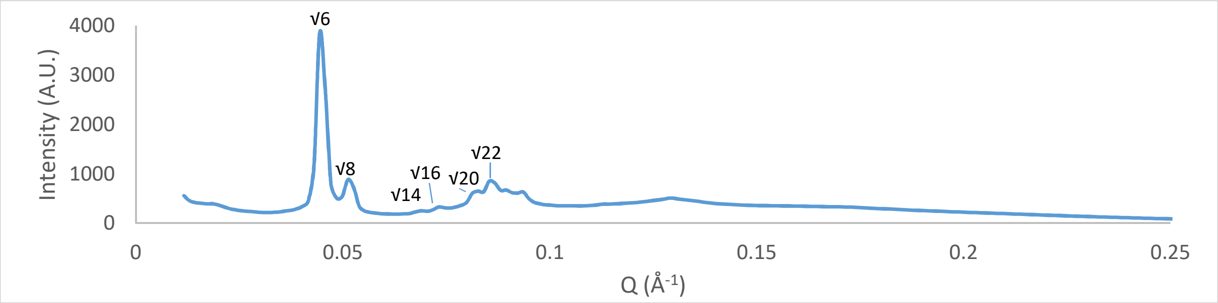

(ae) 10%DSPG/MP 70%H2O

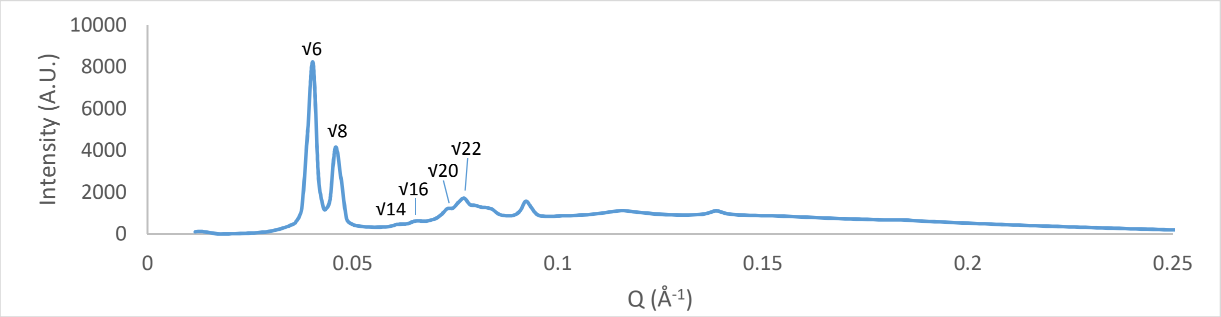

(af) 10%DSPG/MP 75%H2O (mix phase: L_α(*))

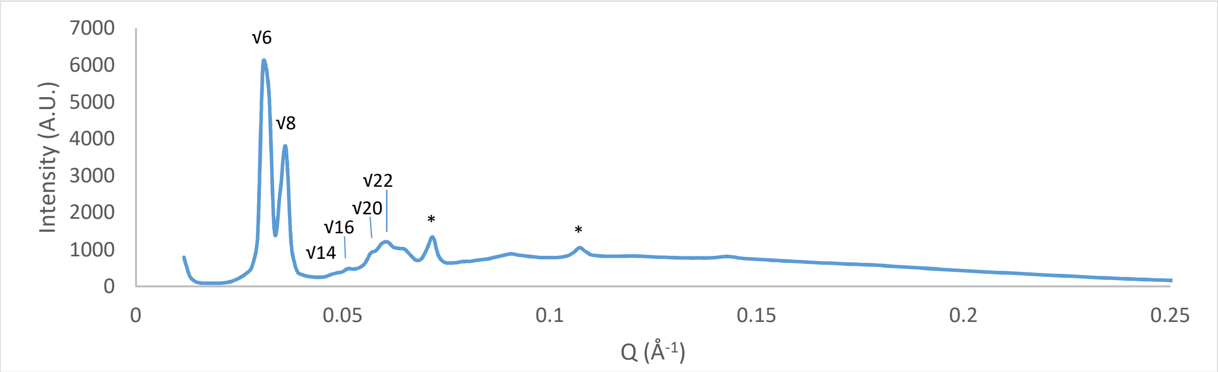

(ag) 10%DSPG/MP 80%H2O (mix phase: L_α(*))

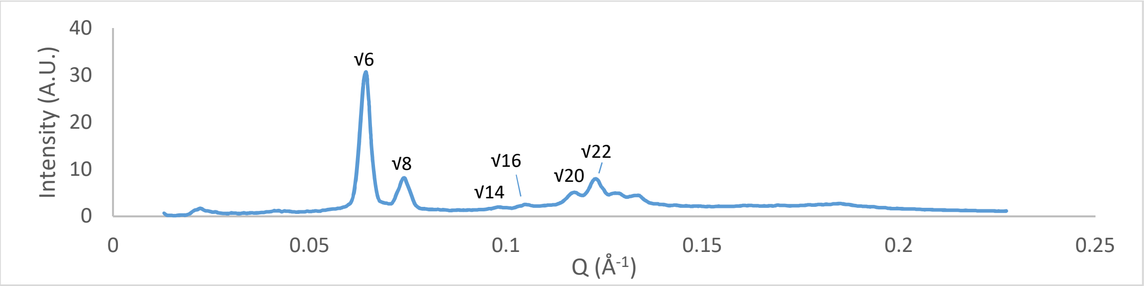

(ah) 11%DSPG/MP 60%H2O

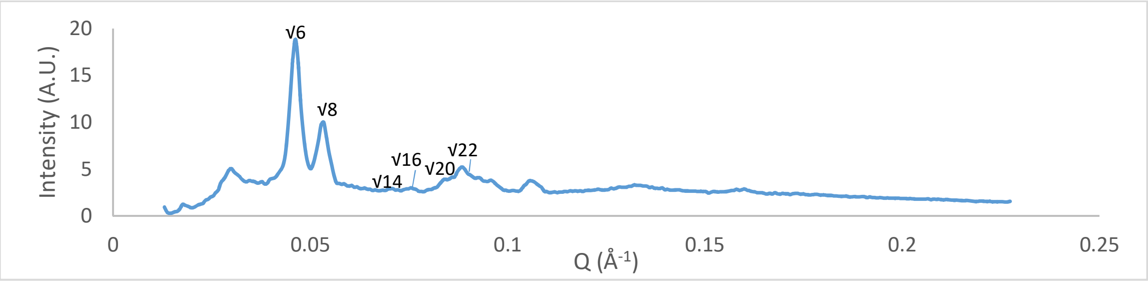

(ai) 11%DSPG/MP 70%H2O (mix phase)

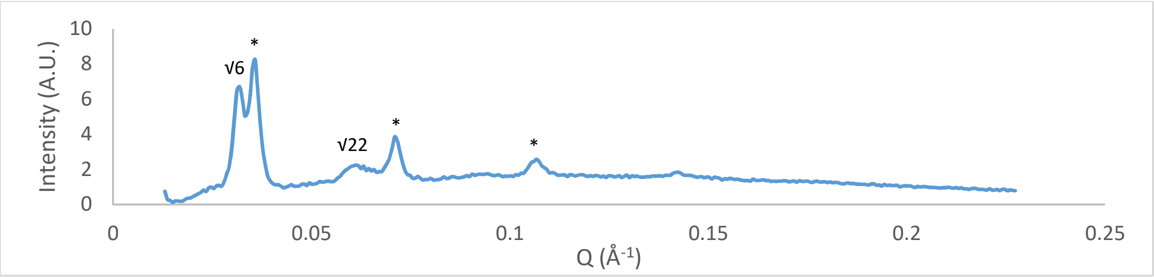

(aj) 11%DSPG/MP 80%H2O (mix phase: L_α(*))

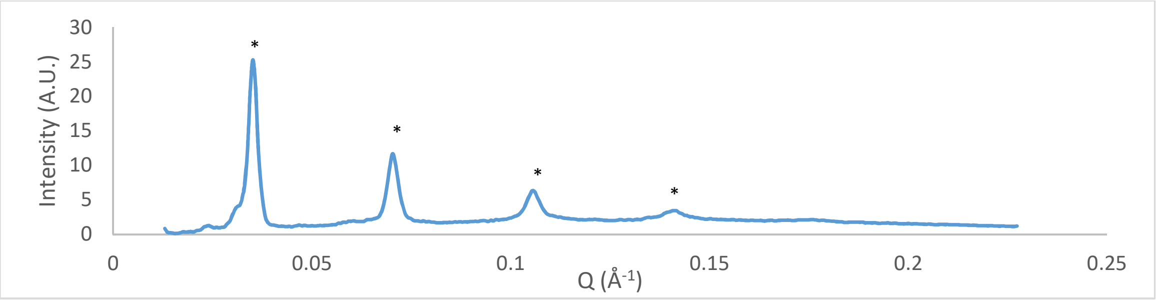

(ak) 12%DSPG/MP 80%H2O - L_α peaks (*)

Supplementary Figure 1 (a)-(ak): 2D SAXS spectra of scattering intensities plotted versus the scattering vector q, of the monopalmitolein:DSPG:water system at various hydration levels (e.g. ranging from 40% to 80% hydration). The observed peaks in a spacing ratio √6: √8: √14: √16: √20: √22 are indicative of a double gyroid cubic symmetry. The observed peaks in a spacing ratio √2: √4: √6: √10: √12: √14 are indicative of a primitive cubic symmetry.

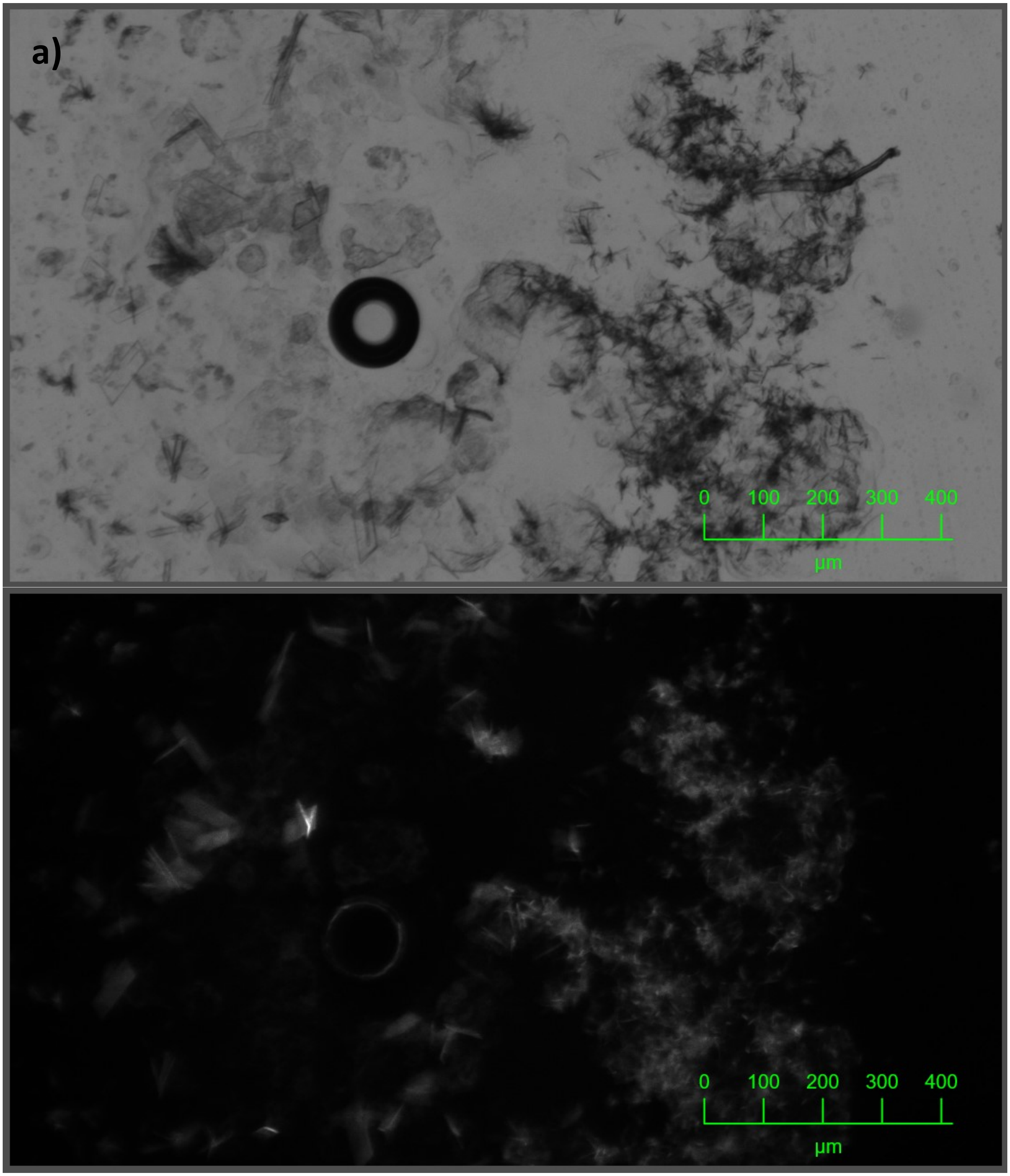
a)
0 100 200 300 400
µm
0 100 200 300 400
µm

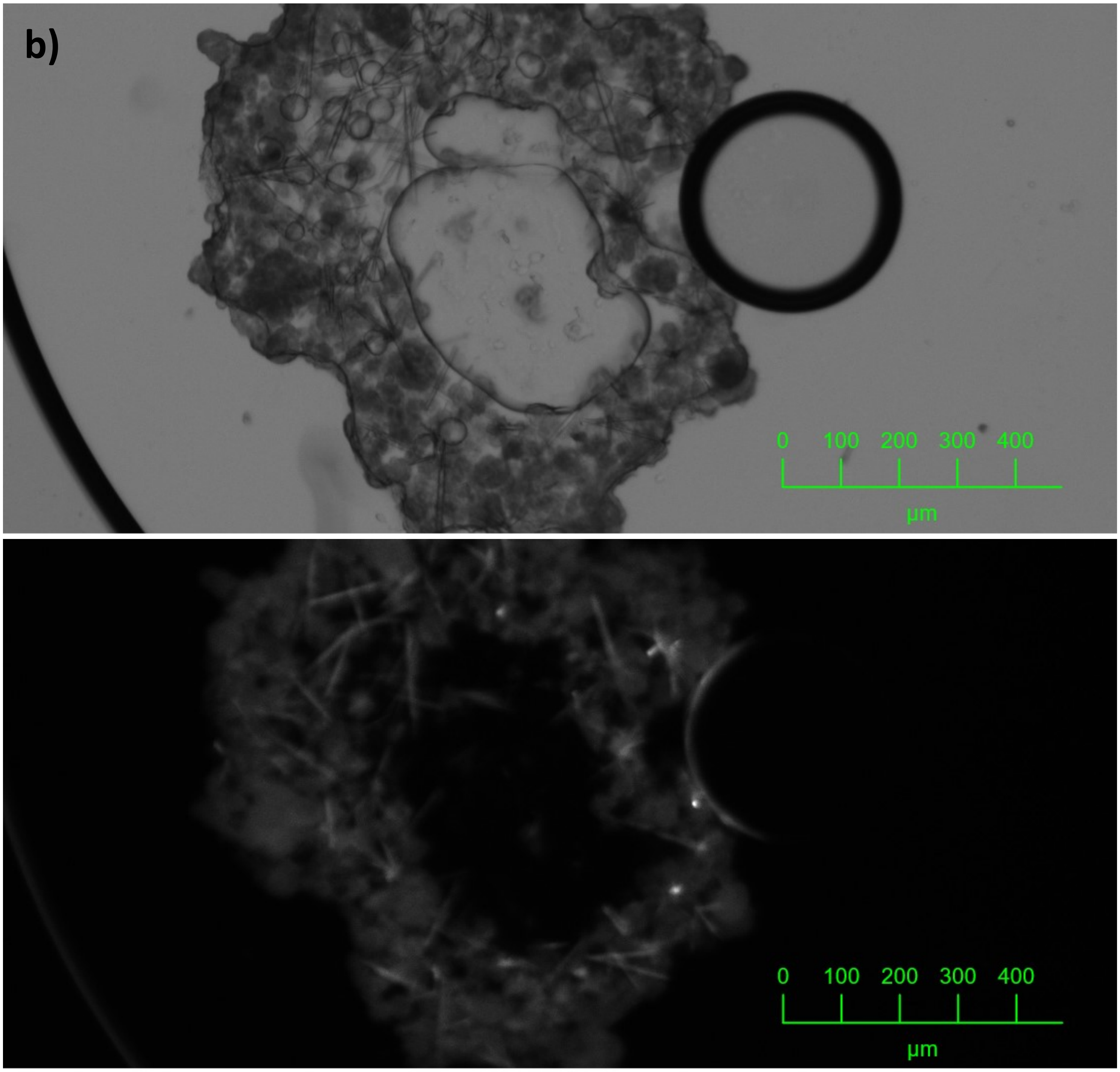

Supplementary Figure 2: Intimin crystals grown in (a) 5%DSPG/MP (Top: Condenser Image, Bottom: UV Image) **and (b) 10%DSPG/MP** (Top: Condenser Image, Bottom: UV Image)

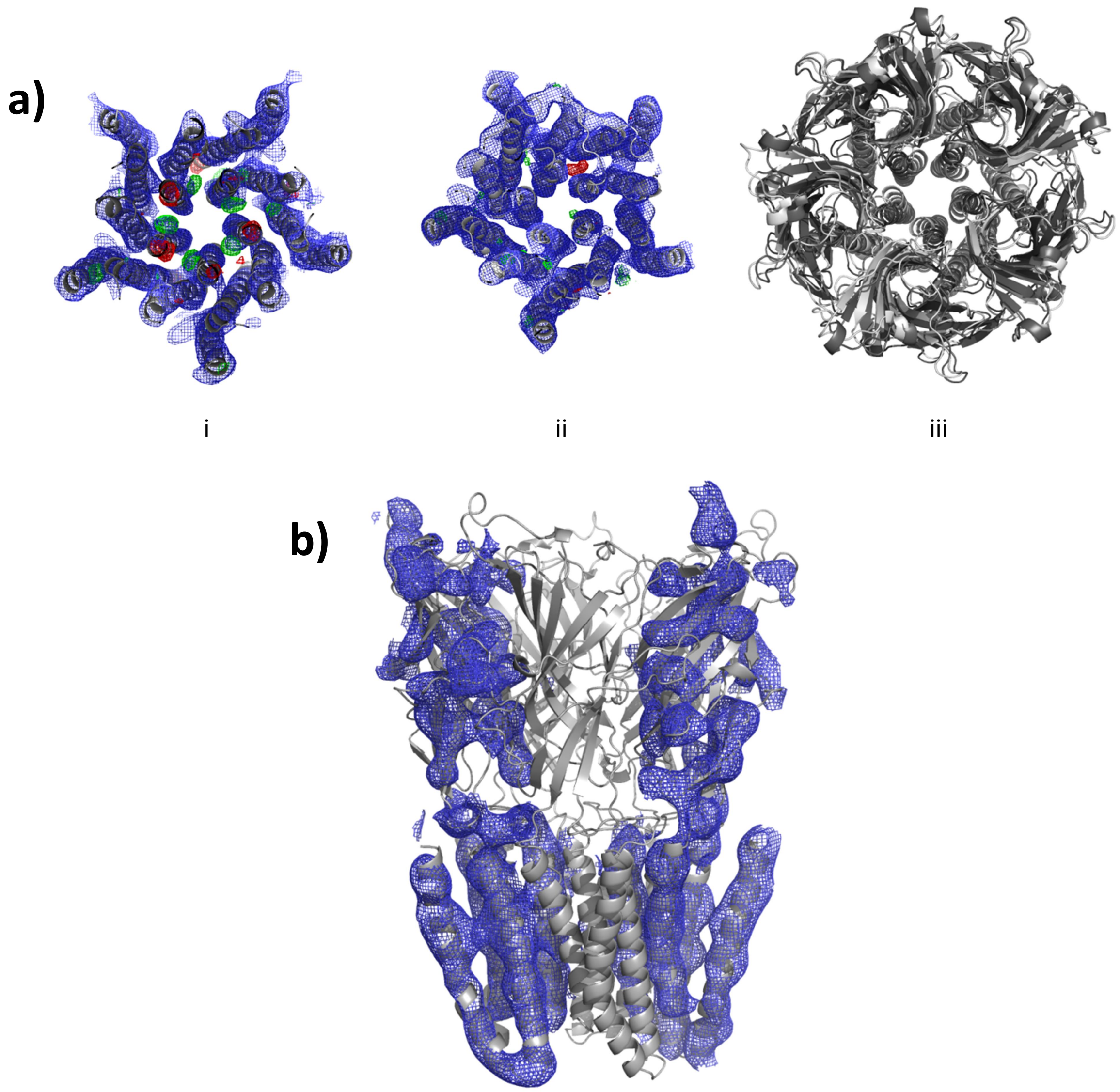

Supplementary Figure 3 (a) Top view on the extracellular domain using either the open form (i) or closed form (ii) (PDB ID 6F7A) as model for the molecular replacement solution and as refinement constraints. The blue map is the 2mFo-DFc map contoured at 1.0σ, while the green and red maps are the respectively positive and negative density difference maps mFo-DFc contoured at 3.0σ. The distribution of residual densities in both maps clearly point to a predominantly closed form due to the position of the inner alpha helixes, as previously reported.[1] For clarity, only the transmembrane domain is shown. (iii) Overlap of the closed (light gray) and open (dark grey) forms. **(b)** Composite omit map 2mFo-DFc, obtained using the so-called "simple" method[2] of the software suite Phenix. For clarity, only the electron density around 2 chains are shown, with a contouring at 1.0σ. In this model-bias-free map, the secondary structure of the protein remains very well resolved in spite of the very low resolution of the dataset, showing the quality of the phasing solution.

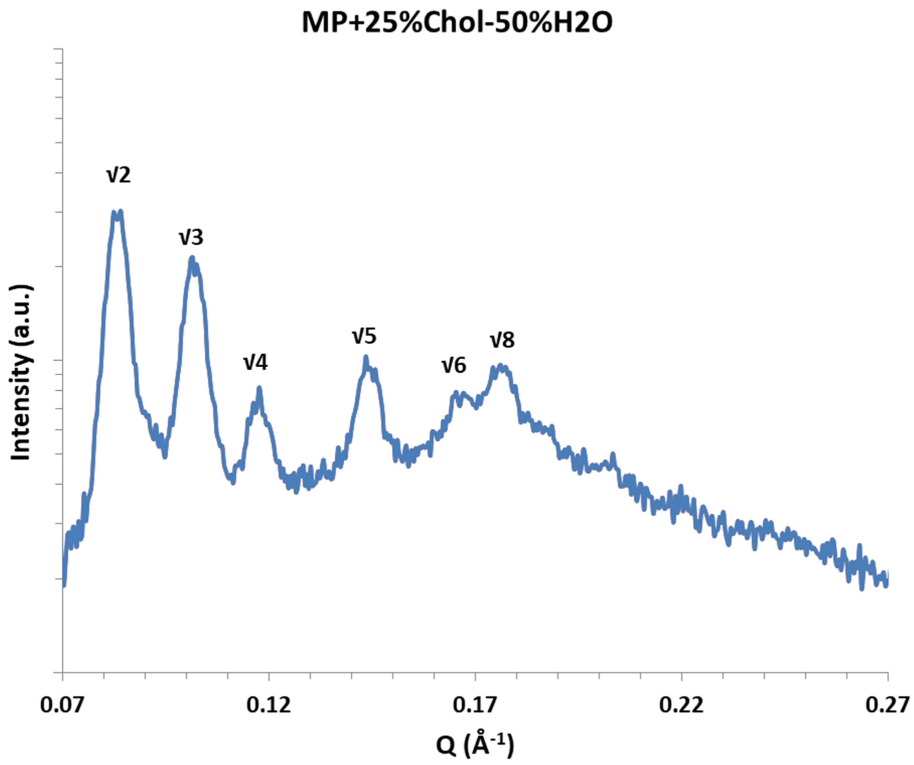

Supplementary Figure 4: 2D SAXS spectra of scattering intensities plotted versus the scattering vector q, of the monopalmitolein:cholesterol:water system at 50% hydration. The observed peaks are in a spacing ratio √2: √3: √4: √6: √8: √9 indicative of a double diamond cubic symmetry.

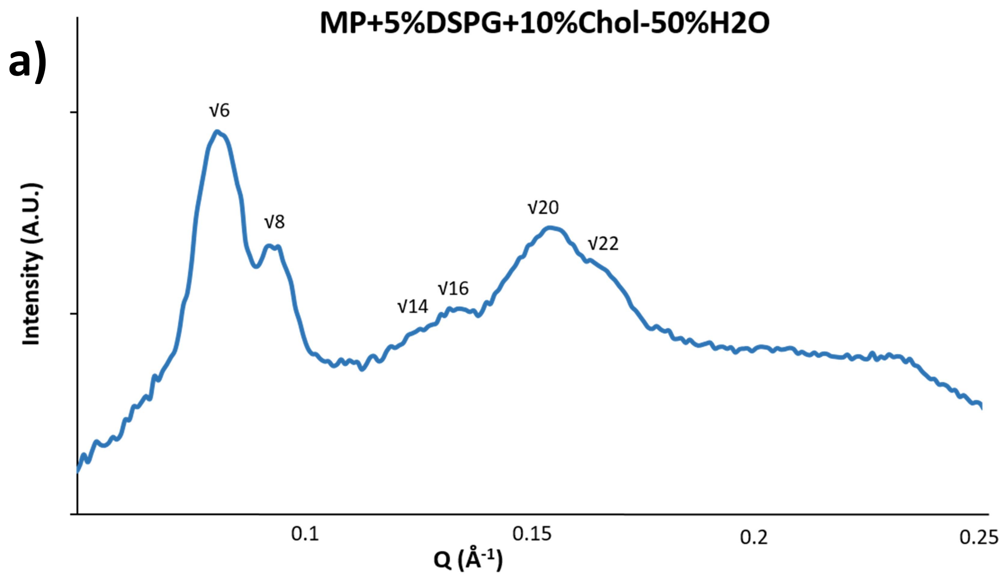

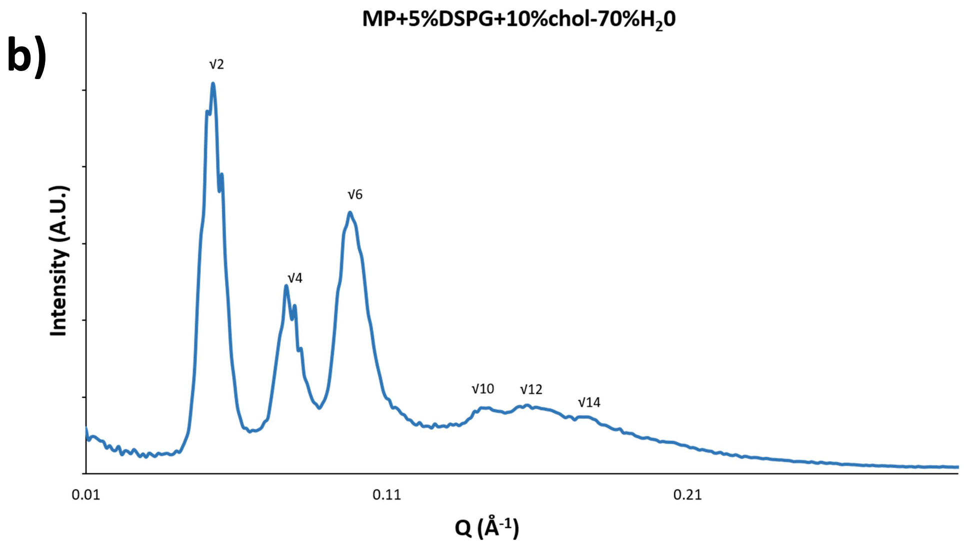

Supplementary Figure 5 (a): 2D SAXS spectra of scattering intensities plotted versus the scattering vector q, of the monopalmitolein:DSPG:cholesterol:water system at 50% hydration. The observed peaks are in a spacing ratio √6: √8: √14: √16: √20: √22 indicative of a double gyroid cubic symmetry. **(b):** 2D SAXS spectra of scattering intensities plotted versus the scattering vector q, of the monopalmitolein:DSPG:cholesterol:water system at 70% hydration. The observed peaks are in a spacing ratio √2: √4: √6: √10:√12:√14 indicative of a primitive cubic symmetry.

Supplementary Table 1 - GLIC crystals diffraction data (PDB ID 6F7A)

	GLIC (PDB ID 6F7A)
Wavelength	1
Resolution range	48.2 - 6.001 (6.215 - 6.001)
Space group	C 2 2 21
Unit cell	75.94 208.22 255.29 90 90 90
Total reflections	28508
Unique reflections	5300 (533)
Multiplicity	5.4
Completeness (%)	0.98
Mean I/sigma(I)	4.99 (0.94)
Wilson B-factor	284.47
R-meas (%)	41.9 (325)
CC1/2	98.1 (13.1)
Reflections used in refinement	5287 (533)
Reflections used for R-free	265 (26)
R-work	0.2861 (0.3970)
R-free	0.3187 (0.4395)
Number of non-hydrogen atoms	11682
macromolecules	11682
Protein residues	1555
RMS(bonds)	0.007
RMS(angles)	0.97
Ramachandran favored (%)	94
Ramachandran allowed (%)	5.3
Ramachandran outliers (%)	0.32
Rotamer outliers (%)	1.2
Clashscore	6.68
Average B-factor	307.36
macromolecules	307.36

Supplementary References

1. Sauguet, L. et al. Crystal structures of a pentameric ligand-gated ion channel provide a mechanism for activation. *Proc. Natl. Acad. Sci. USA* **111**, 966-971 (2014).

2. Afonine, P.V., Moriarty, N.W., Mustyakimov, M., Sobolev, O.V., Terwilliger, T.C., Turk, D., Urzhumtsev, A., and Adams, P.D., FEM: feature-enhanced map. *Acta Cryst.* **D71**, 646-666 (2015).
